# Gut Microbiome Composition in Dystonia Patients

**DOI:** 10.3390/ijms24032383

**Published:** 2023-01-25

**Authors:** Elze R. Timmers, J. Casper Swarte, Ranko Gacesa, Johannes R. Björk, Rinse K. Weersma, Marina A. J. Tijssen, Tom J. de Koning, Hermie J. M. Harmsen, Klary E. Niezen-Koning

**Affiliations:** 1 Department of Neurology, University Medical Center Groningen, University of Groningen, 9700RB Groningen, The Netherlands; 2 Expertise Center Movement Disorders Groningen, University Medical Center Groningen, 9700RB Groningen, The Netherlands; 3 Department of Gastroenterology and Hepatology, University Medical Center Groningen, University of Groningen, 9700RB Groningen, The Netherlands; 4 Department of Clinical Sciences, Lund University, Box 117, 221 00 Lund, Sweden; 5 Department of Genetics, University Medical Center Groningen, University of Groningen, 9700RB Groningen, The Netherlands; 6 Department of Medical Microbiology, University Medical Center Groningen, University of Groningen, 9700RB Groningen, The Netherlands; 7 Laboratory of Metabolic Diseases, Department of Laboratory Medicine, University Medical Center Groningen, University of Groningen, 9700RB Groningen, The Netherlands

**Keywords:** gut microbiome, dystonia, gut–brain axis

## Abstract

Dystonia is a movement disorder in which patients have involuntary abnormal movements or postures. Non-motor symptoms, such as psychiatric symptoms, sleep problems and fatigue, are common. We hypothesise that the gut microbiome might play a role in the pathophysiology of the (non-)motor symptoms in dystonia via the gut–brain axis. This exploratory study investigates the composition of the gut microbiome in dystonia patients compared to healthy controls. Furthermore, the abundance of neuro-active metabolic pathways, which might be implicated in the (non-)motor symptoms, was investigated. We performed both metagenomic and 16S rRNA sequencing on the stool samples of three subtypes of dystonia (27 cervical dystonia, 20 dopa-responsive dystonia and 24 myoclonus-dystonia patients) and 25 controls. While microbiome alpha and beta diversity was not different between dystonia patients and controls, dystonia patients had higher abundances of *Ruminococcus torques* and *Dorea formicigenerans*, and a lower abundance of *Butyrivibrio crossotus* compared to controls. For those with dystonia, non-motor symptoms and the levels of neurotransmitters in plasma explained the variance in the gut microbiome composition. Several neuro-active metabolic pathways, especially tryptophan degradation, were less abundant in the dystonia patients compared to controls. This suggest that the gut–brain axis might be involved in the pathophysiology of dystonia. Further studies are necessary to confirm our preliminary findings.

## 1. Introduction

Dystonia is a hyperkinetic movement disorder characterised by sustained or repetitive involuntary muscle contractions resulting in abnormal movements or postures [1]. Next to these motor symptoms, many patients with dystonia suffer from non-motor symptoms, such as psychiatric symptoms, sleep problems, fatigue and pain [2,3]. While these non-motor symptoms are considered to be part of the phenotype, the exact pathophysiology of both the motor and the non-motor symptoms in dystonia is not yet fully elucidated. Dystonia is considered a network disorder with multiple brain areas involved. Communication between the different regions involved in the brain motor network mainly depends on neurotransmitters and a role of the dopaminergic, serotonergic and noradrenergic systems is suspected in the pathophysiology of dystonia [4,5]. The most important neurotransmitter involved in dystonia is considered to be dopamine, since the direct and indirect output pathways of one of the implicated brain areas, the basal ganglia, are closely regulated by dopamine. Alterations in the concentrations of dopaminergic metabolites or receptors have been described in many types of dystonia [4]. Furthermore, a role of serotonin in dystonia is suggested, based on the differences in metabolites of serotonin in the cerebrospinal fluid, but also because of the fact that serotonergic drugs can induce dystonia [5]. These neurotransmitter systems can be influenced by several factors, including the gut microbiome.

Lately, the role of the gut microbiome in several neurological and psychiatric diseases via the gut–brain axis has gained attention [6,7]. Most of the existing knowledge on the gut–brain axis comes from experiments in germ-free animal models [8,9]. In the last few years, more studies on the human gut microbiome of patients have been conducted with both neurological and psychiatric disorders [10,11,12,13]. In patients with movement disorders, especially Parkinson’s disease, alterations in the composition of the gut microbiome have been observed [14]. However, the role of the gut microbiome in dystonia patients remains largely unknown. Interestingly, in a case study, faecal microbiota transplantation led to an improvement of chronic diarrhoea and dystonic symptoms in a woman with myoclonus dystonia (MD) [15]. Furthermore, Ma et al. studied the gut microbiome of 57 dystonia patients using 16S rRNA sequencing and found an increased abundance of *Closteridiales* and a decreased abundance of some *Bacteriodes* species compared to healthy controls [16]. These studies suggest that the gut microbiome potentially has a role in the pathogenesis of dystonia and the severity of symptoms. This is not only the case for the motor symptoms; the gut microbiome might also play a role in the high prevalence of non-motor symptoms observed in patients with dystonia as it has been previously shown that alterations in the gut microbiome are linked to depression and anxiety in studies with humans and animal models [17].

The gut–microbiome axis encompasses the bidirectional interaction between the gut, including its microbiome and the brain. The four major routes which are involved in the gut–brain axis are: (1) the hypothalamic–pituitary–adrenal (HPA) axis, (2) the immune system, (3) the vagus nerve or afferent spinal nerves and (4) microbial- and host-derived products including neuropeptides and neurotransmitters [18], the latter being the main focus of this study, since the neurotransmitters serotonin and dopamine are implicated in dystonia [4,5]. The microorganisms residing in the gut can not only produce these neurotransmitters themselves, but are also known to be involved in the regulation of several neurotransmitter systems of the host [19]. Multiple studies focused on serotonin, especially the tryptophan (the precursor of serotonin) metabolism [20,21]. There is accumulating evidence that serotonin functions as a key neurotransmitter both in the central nervous system and in the gastrointestinal tract and, therefore, acts as an important regulator in the gut–brain axis. In a previous study, a low level of tryptophan was found in the plasma of dystonia patients compared to healthy controls [5,22].

We hypothesise that the gut microbiome might play a role in the pathophysiology of the motor and non-motor symptoms in dystonia patients via neuro-active metabolic pathways. This exploratory study investigates the composition of the gut microbiome and the abundance of metabolic pathways in dystonia patients compared to healthy controls. We performed both metagenomic and 16S rRNA sequencing on the stool samples of three different subtypes of dystonia and healthy controls. We included idiopathic cervical dystonia (CD) patients, the most common type of dystonia, and two genetically confirmed dystonia groups, dopa-responsive dystonia (DRD) and MD. In these dystonia subtypes, the severity of the motor symptoms differs between patients, and we showed in previous studies in this cohort that non-motor symptoms are highly present [23,24,25]. The first aim of this study was to characterise the composition of the gut microbiome in dystonia patients and compare this with the gut microbiome of healthy controls. Next, we wanted to investigate its relation with the motor and non-motor symptoms and gain insight into the abundance of neurotransmitter-producing or -regulating species and metabolic pathways involved.

## 2. Results

### 2.1. Clinical Characteristics and Non-Motor Symptoms

The clinical characteristics of participants are described in Table 1. Sex, BMI and the Bristol stool chart score were not significantly different between all four groups. Patients with DRD and MD were relatively younger than the CD patients and healthy controls, but this was not statistically significant (*p* = 0.07). Significantly more CD patients (41%) smoked than the other groups (10%, 17%, 24%, *p* = 0.02).

We did not find any differences in the use of medication which are known to influence the gut microbiome, such as proton-pump inhibitors, metformin and laxatives (Table 1). Most of the DRD patients used levodopa (75%) and in the CD and MD groups benzodiazepines were frequently (22% and 25%, resp.) used to minimise the dystonic symptoms.

Psychiatric disorders, sleep disturbances and fatigue were more common in the dystonia groups compared to the controls (Table 1).

### 2.2. The Diversity of the Gut Microbiome of Dystonia Patients Is Not Significantly Different from Healthy Controls

**Figure 1:**
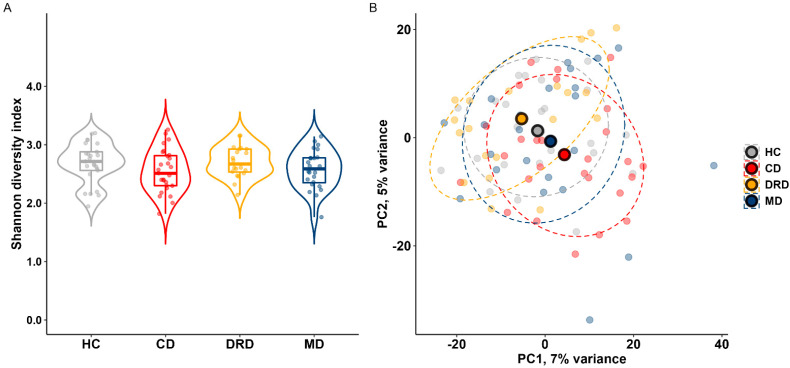
(**A**). violin plot depicting the Shannon diversity index, a quantitative measure for the number and abundance of species within a sample. We found no significant differences in Shannon diversity index (*p* > 0.05). (**B**). Principal component plot based on Aitchison distance for dystonia patients and healthy controls. PC: principal coordinate; HC: healthy controls; CD: cervical dystonia; DRD: dopa-responsive dystonia; MD: myoclonus dystonia.

To characterise the gut microbiome of dystonia patients, we first analysed alpha and beta diversity from the data obtained from metagenomic sequencing. The Shannon diversity index, a quantitative measure for the number and abundance of species within a sample was not significantly different between the different dystonia subtypes (*p* > 0.05, Figure 1A), nor between the whole dystonia group compared to healthy controls (*p* > 0.05). The composition of the gut microbiome on the species level was visualised using principal component analysis (PCA) based on Aitchison distances. No significant differences between the whole dystonia group and healthy controls were found (*p* > 0.05). When comparing dystonia subtypes and healthy controls, a significant difference for PC1 and PC2 between CD and DRD patients (Wilcoxon test: *p* = 0.01; *p* = 0.03) and for PC4 between MD patients and healthy controls were found (Wilcoxon test: *p* = 0.04) (Figure 1 and Supplementary Figure S1). However, PC1, PC2 and PC4 explained only a small part of the variance (7%, 5% and 3%, respectively).

### 2.3. Distinct Gut Microbial Features of Dystonia Patients Compared to Healthy Controls

**Figure 2:**
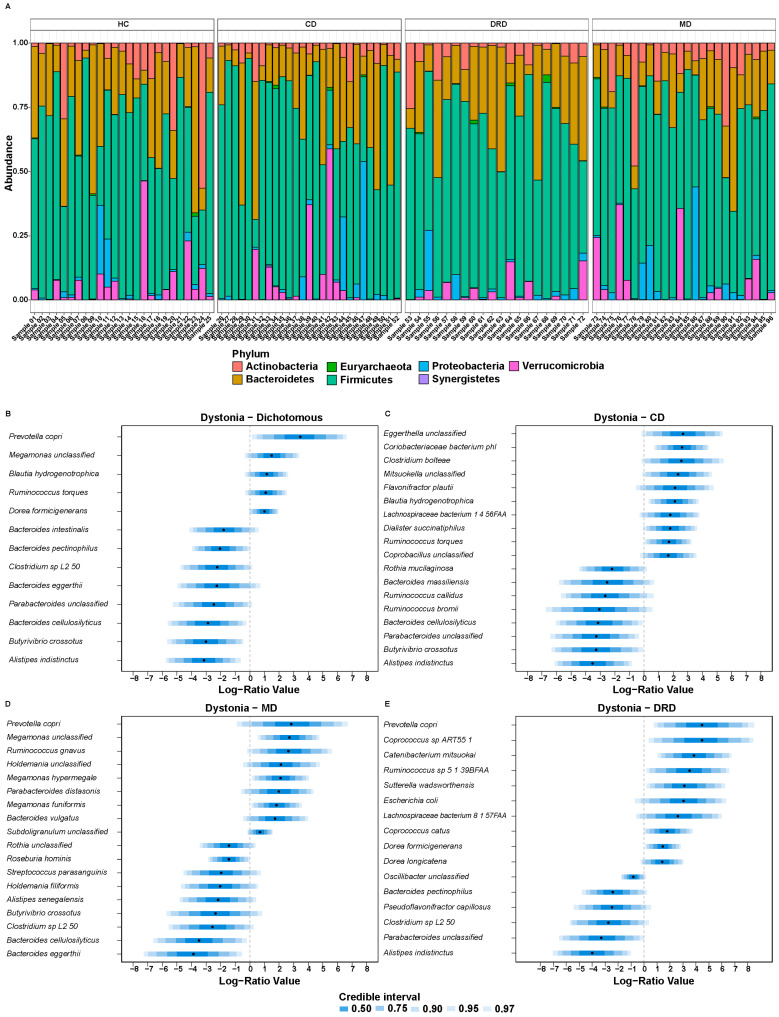
(**A**). Phylum barplot for dystonia patients and healthy controls. (**B**–**E**). Results of a *pibble* model showing differences in species level relative abundance between dystonia patients and healthy controls with a 90% credible interval cut-off. Plots show the comparison between (**B**) the whole dystonia group and the healthy controls, (**C**) cervical dystonia (CD) and healthy controls, (**C**) dopa-responsive dystonia (DRD) and healthy controls and (**E**) myoclonus dystonia (MD) compared to healthy controls.

We did not observe any differences on the phylum level between dystonia patients and healthy controls (Figure 2A). On the species level, higher abundances of *Prevotella copri, Megamonas unclassified, Ruminococcus torques* and *Dorea formicigenerans,* and lower abundances of several *Bacteroides* species and *Butyrivibrio crossotus* were found in the whole dystonia group compared to controls (Figure 2B). Next, we compared each subtype of dystonia to healthy controls. CD patients were characterised by higher abundances of *Eggerthella, Coriobacteriaceae bacterium phi, Dialister succinatiphilus* and *R. torques* which were found, and lower abundances of *Bacteroides massiliensis* and *Butyrivibrio crossotus (phylum: Firmicutes)* compared to healthy controls (Figure 2C). MD patients were characterised by higher abundances of *P. copri, Ruminococcus gnavus* and *Megamonas hypermegale*, and lower abundances of *Bacteroides eggerthii, Bacteroides cellulosilyticus* and *Clostridium sp. L2 50* (Figure 2D). Finally, DRD patients exhibited higher abundances of *P. copri, Sutterella wadsworthensis, Escherichia coli* and *D. formicigenerans,* and lower abundances of *Bacteroides pectinophilus* (Figure 2E).

The metagenomics and 16S rRNA findings were consistent (Figure 2C, Supplementary Figure S2 and Supplementary results).

### 2.4. Association with Clinical Characteristics and Non-Motor Symptoms

**Figure 3:**
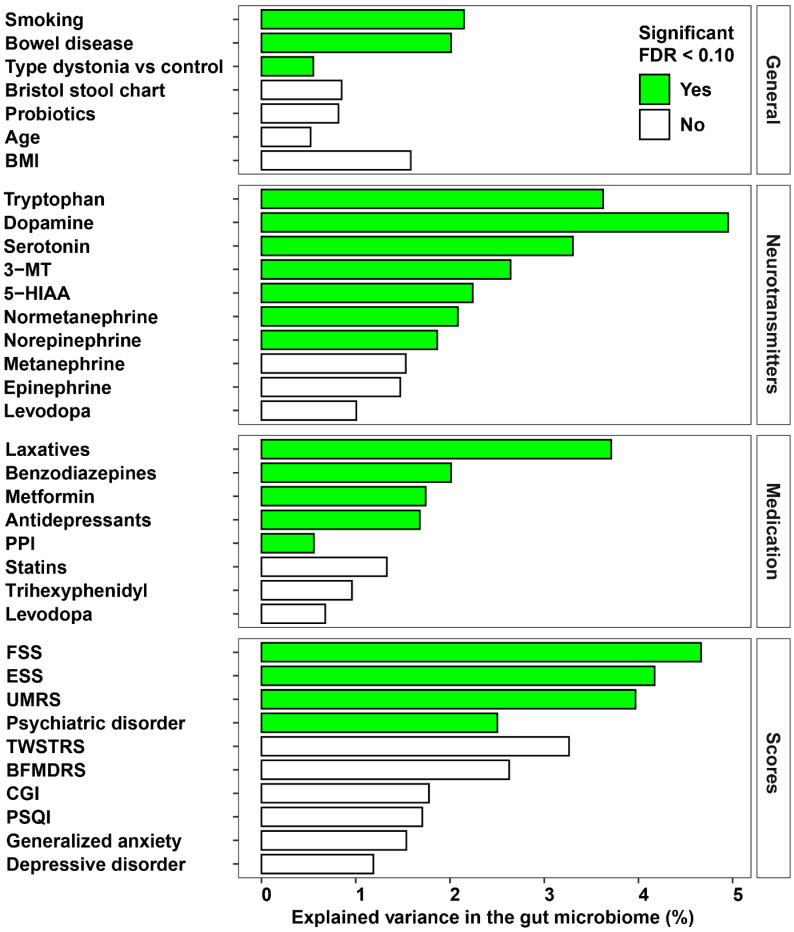
Results of the permutational multivariate analysis of variance using distance matrices (PERMANOVA) to assess the explained variance in the Aitchison distance matrix.

A permutational multivariate analysis of variance (PERMANOVA) on the Aitchison distance revealed that dystonia explained 0.54% of the variance in community composition (*FDR =* 8.4 × 10^−3^). Baseline characteristics, such as having a bowel disease in your medical history (mainly irritable bowel syndrome) and smoking, significantly explained 2.01% and 2.15% of the variance (*FDR* < 0.05), respectively. Several medications, including laxatives, benzodiazepines, metformin, antidepressants and PPI, significantly explained a part of the variance in the community composition as well (*FDR <* 0.10, Figure 3). The severity of dystonic symptoms itself (BFMDRS) did not explain a significant part of the variance, while the UMRS, a score reflecting the severity of myoclonic jerks in dystonia patients, was statistically significant (explained variance of 3.97%, *FDR* < 0.01). The presence of a lifetime psychiatric disorder and scores reflecting fatigue and excessive daytime sleepiness (FSS and ESS) significantly explained 2.50%, 4.67% and 4.17% of the variance (*FDR <* 0.05) in the community composition, respectively. Furthermore, concentrations of tryptophan, serotonin, 5-hydroxyindoleacetic acid (5-HIAA), dopamine, 3-methoxytyramine (3-MT), normetanephrine and norepinephrine significantly explained variance in the gut microbiome composition (*FDR <* 0.10)

### 2.5. Metabolic Pathways

**Figure 4:**
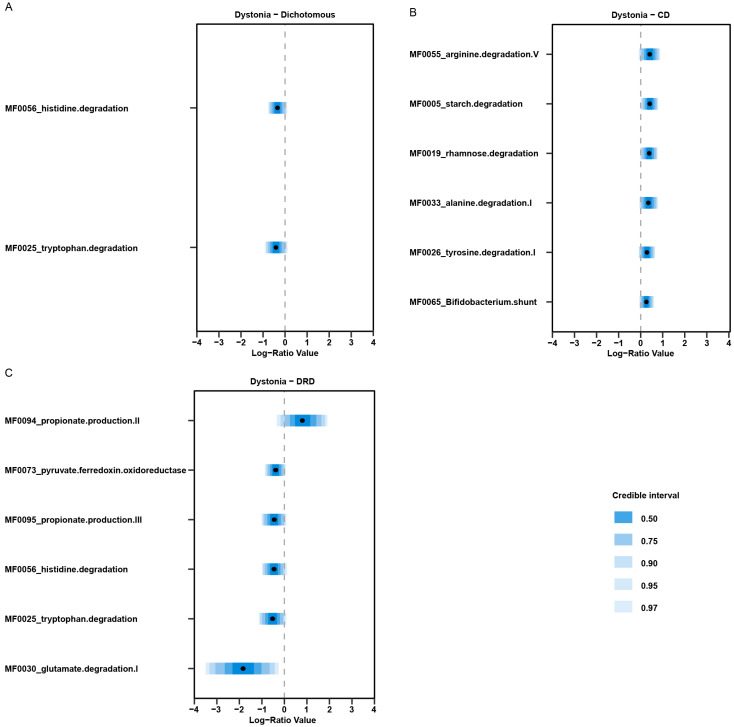
Results of the *pibble* model showing differences in abundance of neuro-active metabolic pathways between dystonia patients and healthy controls based on results of the metagenomic sequencing. A cut-off of 90% confidence interval was used. Plots show the comparison between (**A**) healthy controls and the whole dystonia group, (**B**) cervical dystonia (CD) and (**C**) dopa-responsive dystonia (DRD) as depicted. We did not find any results for myoclonus dystonia (MD) compared with healthy controls.

We next analysed microbial metabolism and the neuroactive potential of the gut microbiome of dystonia patients. The total number of detected metabolic pathways (the richness) that was present in the gut microbiota between the groups was significantly different between CD and DRD patients (*p* = 0.04). However, no difference was found between dystonia patients and the healthy controls (*p* > 0.05, Supplementary Figure S3). We detected several differentially abundant metabolic pathways between dystonia and healthy controls, for example, tetrapyrrole biosynthesis, the superpathway of glucose and xylose degeneration and gluconeogenesis (Supplementary Figure S4). Since we were especially interested in neuro-active pathways, a separate analysis was performed with only the pathways that are known to be neuro-active. We used the approach reported by Valles-Colomer et al. to reclassify the KEGG orthologs into neuroactive modules [10,26]. A lower abundance of the histidine and tryptophan degeneration pathway was found in dystonia patients compared to controls (Figure 4).

## 3. Discussion

In this exploratory study we investigated the composition of the gut microbiome in dystonia patients and correlated it to clinical characteristics. The species diversity of the microbiome within and between samples (alpha diversity and beta diversity, respectively) was not different between dystonia patients and controls (Figure 1). However, we did find several microbial species and pathways that were enriched in the dystonia patients compared to healthy controls (Figure 2). Having a diagnosis of dystonia, the non-motor symptoms and measurements of levels of mono-amine neurotransmitters significantly explained variation in the gut microbiome composition (Figure 3). Several neuro-active metabolic pathways, especially histidine and tryptophan degradation, were less abundant in the dystonia group compared to controls (Figure 4).

The results of our study did not show a difference in the alpha diversity between dystonia patients and controls. This was similar to the results of the only previous study that investigated the gut microbiome in dystonia patients. However, the study of Ma et al. showed a difference in the beta diversity with an increased abundance of *Clostridiales* and decreased abundance of *Bacteroidetes* [16]. Although the beta diversity was not different in our study, several *Clostridiales* sp., such as *B. hydrogenotrophica, R. torques* and *D. formicigenerans*, were more abundant in dystonia patients than in the healthy controls. In our cohort, many species belonging to the *Bacteriodetes* phylum were less abundant as well, except for *P. copri*, which was more abundant in the whole dystonia group and in the MD and DRD cohorts. This species was previously associated with gut inflammation and several adverse conditions such as insulin resistance, hypertension and rheumatoid arthritis [27,28,29]. Surprisingly, in other neurological and psychiatric disorders, such as Parkinson’s disease, multiple sclerosis and depression, the *Prevotellaceae* family was less abundant [12,13,30]. The differences between our results and the findings of the study of Ma et al. might be explained by methodological aspects and differences between the cohorts [16]. Ma et al. Used 16S rRNA sequencing, while in our study, we used both 16S rRNA as the metagenomic sequencing methods. Furthermore, the ethnicity of our cohorts was different and the dystonia groups in our cohort were more homogenous.

The results of our study show evidence of a dysbiosis of the gut microbiota in dystonia patients with a shift of strictly anaerobic bacteria to more aerotolerant bacteria, such as *Eggerthella, Coriobacteriaceae bacterium phi, R. torques, R. gnavus* and *D. formicigenerans.* These potentially harmful bacteria were found to be increased in previous studies in patients with multiple sclerosis and persons who had a disrupted circadian rhythm due to night shifts [31,32]. Furthermore, some pathogenic bacteria, such as the small intestine bacteria *Megamonas unclassified* and *Dialister succinatiphilus*, were more abundant in the dystonia groups. In the DRD group, *E. coli* and *S. wadsworthensis*, both bacteria associated with gastrointestinal diseases, were more common, further suggesting a dysbiosis in dystonia patients [33,34].

The next aim of this study was to analyze the relationship between the motor and non-motor symptoms of dystonia patients and the composition of the gut microbiome. First, having a diagnosis of dystonia significantly explains a significant proportion of the variance in the composition of the gut microbiome, suggesting that there might be a link between the gut microbiome and dystonia. Although we did not find an association with the severity of dystonia, the severity of the myoclonic jerks (mainly in the CD and MD group) was associated with the composition of the gut microbiome. This might be an explanation of the beneficial effect of a faecal microbiota transplantation in a patient with MD [15]. The non-motor symptoms, such as having a psychiatric diagnosis and the severity of fatigue and sleepiness, significantly predicted some of the variance of the composition of the microbiome. This is in line with previous studies which showed a connection between psychiatry and the gut microbiome [17].

Comparing our results of the metagenomic sequencing with 16S rRNA sequencing on a genus level showed that there was an increased abundance of *Dorea* and a decreased abundance of *Butyrivibrio. Dorea* and *Butyrivibrio* contain bacteria species that are known to produce short-chain fatty acids (SCFA), such as proprionate, acetate and butyrate. SCFAs play an important role in the gut homeostasis and are thought to reduce the inflammatory properties of immune cells [35,36]. In Parkinson’s disease, several studies showed that there is a lower abundance of these SCFAs, which are thought to contribute to the pathophysiology [37,38]. *Dorea* are acetate- and proprionate-producing bacteria, while *Butyrivibrio* are known to produce butyrate [39,40]. In dystonia, there might be a shift from butyrate-producing bacteria (such as *Butyrivibrio*) towards proprionate-producing bacteria (such as *Dorea*). Although we did not directly measure the SCFAs, this hypothesis is supported by the higher abundance of the metabolic pathway of proprionate production in DRD patients. Future studies examining SCFAs in dystonia patients might further shed light on their role in the pathophysiology of dystonia.

Another mechanism that might be involved is the serotonin and tryptophan metabolism. Serotonin is a neurotransmitter suggested to be involved in dystonia [5]. Around 90% of the serotonin in the body is produced in the gut from the essential amino-acid tryptophan, which is mainly derived from diet. This indicates that the biological availability of tryptophan in the body mainly depends on the metabolisation of tryptophan in the gut. An animal study showed that spore-forming bacteria can influence host serotonin metabolism [41]. Several other bacteria, such as *Lactobacillus* spp., *E. coli* and *Clostridium sporogenes,* are known to play a role in the gut–brain axis by metabolising tryptophan into several molecules including neuro-active substances, such as kynurenic acid and quinolinic acid [20,21]. Surprisingly, in our study, we found a decrease in the tryptophan degeneration pathway in dystonia patients compared to controls. In line with this, there was a lower abundance of *Bacteroides eggerthii* in dystonia patients. This species is known to metabolise tryptophan into skatole and indole-3-acetic acid [42]. In contrast to our findings, in the study of Ma et al., an increase in the tryptophan degeneration pathway was reported. This difference might be due to the fact that they predicted the presence of metabolic pathways on the results of the 16S rRNA sequencing, while our results are based on metagenomic sequencing. However, the lower level of tryptophan, that we previously have found in the plasma in the same dystonia cohort, suggests that the tryptophan degradation is increased instead of decreased [22]. It might be that in our cohort, the intake of tryptophan in the diet was lower, resulting in less degradation and lower plasma values. However, together with the finding that levels of tryptophan in the plasma significantly explained some of the variance of the composition of the gut microbiome, it does suggest that the tryptophan metabolism might be affected in dystonia patients. Further research is necessary to confirm our preliminary results.

Our study showed alterations in other neuro-active metabolic pathways as well, including several amino-acid pathways such as histidine, tyrosine and glutamate. The increase in tyrosine degradation that was found in CD patients is of special interest, since tyrosine is the precursor of dopamine. Dopamine is thought to be the main neurotransmitter involved in dystonia and changes in availability might have an effect on dopamine synthesis [4]. Furthermore, we found a decrease of glutamate degradation. Glutamate is an important neurotransmitter implicated in the gut–brain axis and pharmacological activation of glutamate receptors in the cerebellum can induce dystonia in mice [43,44]. Together, these findings show some evidence that the gut–brain axis might also be involved in dystonia.

Our study is one of the first studies investigating the gut microbiome in dystonia patients using metagenomic sequencing data, and our cohorts were well defined and characterised. Although we had a small sample size, which is inevitable with a rare disorder such as dystonia, our findings from metagenomic sequencing and 16S rRNA sequencing were highly consistent. Due to the small sample size and exploratory nature of this study we decided not to correct for possible confounders. Therefore, no definite conclusions can be drawn based on our study, however, our findings can be used as an important guidance for future studies.

## 4. Material and Methods

### 4.1. Study Population

We included three groups of dystonia patients: 27 patients with idiopathic CD; 20 DRD patients with a confirmed guanosine 5′-triphosphate cyclohydrolase 1 gene (*GCH1*) mutation and 24 MD patients with a confirmed epsilon-sarcoglycan gene (*SGCE*) mutation. Twenty-five participants without a movement disorder served as a control group. Missense and frameshift mutations in *GCH1* and *SGCE* were confirmed in different hospitals in the Netherlands using sanger sequencing or next generation sequencing methods. Both children and adults were eligible to participate in order to obtain a sufficient sample size in the two rare genetic forms of dystonia (DRD and MD). In total, 8 children (4 MD and 4 DRD patients) participated in this study. Exclusion criteria were: antibiotic use, diarrhoea or symptoms of stomach flu in the last three months, and healthy controls could not be a first- or second-degree relative of a dystonia patient. All patients were recruited via several medical centres in the Netherlands, and controls via open advertisements or were acquaintances of patients or researchers. Informed consent was obtained from all participants and this study was approved by the medical ethics committee of the University Medical Centre, Groningen (METc 2014/034).

### 4.2. Clinical Characteristics and Non-Motor Symptoms

All subjects participated in a previous study in which data about non-motor symptoms and a blood sample were collected [23,24,25]. Clinical data were already described and included a structured interview and questionnaires, including, but not limited to, medical history, medication use, smoking habits, Bristol stool chart and BMI. In all dystonia patients, the Clinical Global Index (CGI) was used to assess the severity of the movement disorder [45]. Next, depending on the dystonia subtype, the Burke–Fahn–Marsden Dystonia Rating Scale (BFMDRS), the Unified Myoclonus Rating Scale (UMRS) and the Toronto Western Spasmodic Torticollis Rating Scale (TWSTRS) were used [46,47,48].

Age-appropriate standardised questionnaires were used to evaluate presence of psychiatric disorders and severity of depression, anxiety, obsessive compulsive disorder (OCD), daytime sleepiness, fatigue and quality of sleep (for detailed information see supplementary Table S1). Participants underwent a venous puncture to obtain a blood sample which was stored until analysis at −80 ºC. Metabolites of the serotonergic, dopaminergic and adrenergic system were measured using an on-line solid-phase extraction–liquid chromatographic method with tandem mass spectrometric detection (LC-MS/MS), as has been described previously [49,50]

### 4.3. Collection of Faecal Samples

All participants collected a faecal sample at home, and were instructed to store the faeces immediately after obtaining the sample in their home freezer. The samples were collected and shipped to the UMCG on solid carbon dioxide. Samples were stored at −20 °C until analysed in one batch.

### 4.4. Analysis of Faecal Microbiota

#### 4.4.1. DNA Extraction

For microbial DNA extraction a double bead-beater procedure was performed, based on Yu et al., 2004, using the QIAamp DNA stool Minikit (Qiagen 51604, Hilden, Germany) [51].

#### 4.4.2. Metagenomic Sequencing

Library preparation was performed using NEBNext^®^ Ultra™ DNA Library Prep Kit for Illumina (total DNA amount < 200 ng) or NEBNext^®^ Ultra™ II DNA Library Prep Kit for Illumina^®^ (total DNA amount > 200 ng). Libraries were prepared according to the manufacturer’s instructions. Metagenomic shotgun sequencing was performed using Illumina HiSeq 2000 sequencing platform. Library preparation and sequencing were performed at Novogene, Cambridge, UK.

#### 4.4.3. 16S rRNA Sequencing

Polymerase chain reaction (PCR) using the TaKaRa Taq Hot start version kit (TaKaRa Bio Inc., Kusatsu, Japan) was used to amplify the genes for the 16S rRNA V4 and V5 region. Primers (341F and 806R) containing a 6-nucleotide Illumina-MiSeq adapter sequence were used and the PCR product was purified with AMPure XP beads (Beckman Coulter, Brea, CA, USA). To ensure equal library presentation for each sample, DNA concentrations were measured with Qubit 2.0 Fluorometer and dilutions were made accordingly. The MiSeq Benchtop Sequencer was used to sequence the normalised DNA library [52].

#### 4.4.4. Metagenomic and 16S rRNA Processing

Processing of the metagenomic data was performed as described by Gacesa et al. and Swarte et al., 2022 [53,54]. In brief: KneadData (version 0.5.1) and Bowtie2 (version 2.3.4.1) were used to remove low-quality reads and reads aligned to the human genome. Taxonomy alignment was performed by MetaPhIAn2 (version 2.72) and Metacyc pathways were profiled by HUMAnN2 (version 0.11.1). Analyses were performed using locally installed tools and databases at UMCG and University of Groningen (RUG). For the 16S rRNA data, PAired-eND Assembler for DNA sequences (PANDAseq) was used to increase the quality of sequenced reads and readouts with a quality score lower than 0.9 were discarded. Quantitative Insights Into Microbial Ecology (QIIME) was used to assign taxonomy to the phylum, class, order, family and genus level [52]. After quality control and filtering for a relative abundance of at least 1% and a prevalence of 10% across samples, we retained a total of 363 taxa (7 phyla, 13 class, 17 order, 33 family, 78 genera and 215 species) and 351 metabolic pathways in the metagenomic sequencing data and a total of 148 taxa (6 phyla, 8 class, 19 order, 31 family, 84 genera) in the 16S rRNA sequencing data.

### 4.5. Statistical Analysis

All baseline data were quantitively described. Kruskal–Wallis, χ^2^-test or Fisher–Freeman–Halton exact test were used to determine differences in clinical characteristics and non-motor symptoms between cases and controls. Analyses were performed in IBM SPSS Statistics version 28, or in R version 3.6. A *p*-value < 0.05 was considered statistically significant.

To calculate microbiome alpha diversity, we used the Shannon diversity index which was calculated using QIIME microbiome analysis software. Beta diversity was computed using the Aitchison distance. Wilcoxon rank sum test was used to determine significant differences in PCs between groups.

We used the Pibble model which implements a bayesian multinomial logistic-normal model to analyse and identify differentially abundant taxa and metabolic pathways in the gut microbiome between the different dystonia groups and healthy controls [55]. This analysis was performed in R using the *pibble* function from the fido package. We deemed a credible result of 90% not containing zero to be statistically significant. We first analysed the abundance table obtained from the metagenomic sequencing which allowed us to analyse taxa at the species level. After that, we compared those results with our 16S rRNA sequencing data on the *genus* level. The metabolic pathways were assessed only in the metagenomic dataset.

To identify clinical characteristics and non-motor symptoms that significantly explain variance in the gut microbiome, we used permutational multivariate analysis of variance using distance matrices (PERMANOVA) with the *ADONIS* function from the *vegan* package. The Benjamin Hochberg false discovery rate was applied to correct for multiple testing.

## 5. Conclusions

In conclusion, we did not find differences in the alpha and beta diversity of the microbiota in patients with dystonia. However, several microbial species had a different abundance in dystonia patients compared to healthy controls. Some clinical characteristics, especially the non-motor symptoms, were associated with the composition of the gut microbiome. The alterations found in several neuro-active metabolic pathways, including tryptophan degradation, suggest that also in dystonia, the gut–brain axis might be involved in the pathophysiology of dystonia. Further studies, with larger and more homogenous groups, are necessary.

## Figures and Tables

**Table 1:** Demographic and clinical characteristics of participants.

	Cervical Dystonia	Dopa-Responsive Dystonia	Myoclonus-Dystonia	Healthy Controls	*p*-Value
	N = 27	N = 20	N = 24	N = 25	
**Age**	56 (23–73)	48 (15–80)	46 (7–77)	57 (40–86)	*0.07*
**Sex male (%)**	7 (26%)	8 (40%)	9 (38%)	5 (20%)	*0.39*
**Bristol stool chart**	4 (2–6)	4 (1–7)	4 (3–6)	4 (2–6)	*0.10*
**BMI**	25 (19–31)	25 (16–32)	23 (17–31)	24 (21–32)	*0.70*
**Smoking**	11 (41%)	2 (10%)	4 (17%)	6 (24%)	***0.02***
**Use of probiotics**	0 (0%)	1 (5%)	1 (4%)	2 (8%)	*0.53*
**Medication**					
**PPI**	4 (15%)	1 (5%)	3 (13%)	4 (16%)	*0.70*
**Laxative**	1 (4%)	1 (5%)	0 (0%)	2 (8%)	*0.62*
**Metformin**	1 (4%)	0 (0%)	0 (0%)	0 (0%)	*1.00*
**Statin**	5 (19%)	1 (5%)	2 (8%)	6 (24%)	*0.26*
**Benzodiazepine**	6 (22%)	1 (5%)	6 (25%)	1 (4%)	*0.07*
**Trihexyfenidyl**	2 (7%)	0 (0%)	0 (0%)	0 (0%)	*0.25*
**Levodopa**	0 (0%)	15 (75%)	0 (0%)	0 (0%)	***0.00***
**Antidepressants**	0 (0%)	2 (10%)	6 (25%)	1 (4%)	***0.01***
**Antibiotics**	0 (0%)	0 (0%)	0 (0%)	0 (0%)	*1.00*
**Motor symptoms**					
**CGI**	4 (2–7)	2 (1–2)	2 (1–5)	-	***0.00***
**BFMDRS**	-	6 (0–17.5)	6 (0–15)	-	
**UMRS**	6 (0–22)	-	11 (0–62)	-	
**TWSTRS**	14 (10–27)	-	-	-	
**Non-motor symptoms**					
**Psychiatric disorder**	16 (59%)	11 (55%)	16 (67%)	7 (28%)	***0.03***
**Mood disorder**	14 (52%)	7 (35%)	5 (21%)	2 (8%)	***0.01***
**Anxiety disorder**	9 (33%)	10 (50%)	15 (63%)	3 (12%)	***0.00***
**BDI ^1^**	11 (0–28)	4 (0–15)	8 (0–24)	3 (0–19)	***0.01***
**BDI/CDI z-score**	1 (−1–4)	−0.3 (−1–2)	0.2 (−1–4)	−0.4 (−1–3)	***0.00***
**BAI ^1^**	29 (22–46)	27 (21–33)	28 (21–52)	24 (21–42)	***0.02***
**BAI/SCARED z-score**	0.6 (−1–4)	−0.2 (−3–2)	0.3 (−2–6)	−0.5 (−1–4)	***0.01***
**ESS ^1^**	9 (0–24)	10 (1–21)	7.5 (0–17)	4 (0–16)	*0.14*
**FSS ^1^**	37 (12–63)	29 (14–62)	35 (9–62)	23 (9–55)	***0.01***
**PSQI ^1^**	7 (2–16)	8 (1–20)	4 (1–11)	4 (1–15)	***0.03***

Data are presented as median (range) or number (%). All four groups were compared with each other and Kruskal–Wallis tests, χ^2^-test or Fisher–Freeman–Halton exact test were used to compute *p*-values. *p*-values depicted in bold are considered to be statistically significant. PPI: Proton-Pump Inhibitor; CGI: Clinical Global Impression scale; BFMDRS: Burke–Fahn–Marsden Dystonia Rating Scale; UMRS: Unified Myoclonus Rating Scale; BDI: Beck Depression Index; BDI/CDI z-score: z-score of Beck Depression Index (adult questionnaire) and Child Depression Index (child questionnaire); BAI: Beck Anxiety Index; BAI/SCARED z-score: z-score of Beck Anxiety Index (adult questionnaire) and Screen for Child Anxiety Related Emotional Disorders (child questionnaire) ESS: Epworth Sleepiness Scale; FSS: Fatigue Severity Scale; PSQI: Pittsburgh Sleep Quality Index. ^1^ only adult participants.

## Data Availability

The data presented in this study are available on request from the corresponding author. The data are not publicly available due to privacy reasons.

## Supplementary Materials

The following supporting information can be downloaded at: https://www.mdpi.com/article/10.3390/ijms24032383/s1.

## References

[1] Balint B., Mencacci N.E., Valente E.M., Pisani A., Rothwell J., Jankovic J., Vidailhet M., Bhatia K.P. (2018). Dystonia. Nat. Rev. Dis. Prim..

[2] Kuyper D.J., Parra V., Aerts S., Okun M., Kluger B.M. (2011). Nonmotor manifestations of dystonia: A systematic review. Mov. Disord..

[3] Stamelou M., Edwards M.J., Hallett M., Bhatia K.P. (2012). The non-motor syndrome of primary dystonia: Clinical and pathophysiological implications. Brain.

[4] Ribot B., Aupy J., Vidailhet M., Mazère J., Pisani A., Bezard E., Guehl D., Burbaud P. (2019). Dystonia and dopamine: From phenomenology to pathophysiology. Prog. Neurobiol..

[5] Smit M., Bartels A.L., van Faassen M., Kuiper A., Niezen-Koning K.E., Kema I.P., Dierckx R.A., de Koning T.J., Tijssen M.A. (2016). Serotonergic perturbations in dystonia disorders—A systematic review. Neurosci. Biobehav. Rev..

[6] Quigley E.M.M. (2017). Microbiota-Brain-Gut Axis and Neurodegenerative Diseases. Curr. Neurol. Neurosci. Rep..

[7] Ma Q., Xing C., Long W., Wang H.Y., Liu Q., Wang R.-F. (2019). Impact of microbiota on central nervous system and neurological diseases: The gut-brain axis. J. Neuroinflamm..

[8] Mayer E.A., Tillisch K., Gupta A. (2015). Gut/brain axis and the microbiota. J. Clin. Investig..

[9] Cryan J.F., O’Riordan K.J., Cowan C.S.M., Sandhu K.V., Bastiaanssen T.F.S., Boehme M., Codagnone M.G., Cussotto S., Fulling C., Golubeva A.V. (2019). The Microbiota-Gut-Brain Axis. Physiol. Rev..

[10] Valles-Colomer M., Falony G., Darzi Y., Tigchelaar E.F., Wang J., Tito R.Y., Schiweck C., Kurilshikov A., Joossens M., Wijmenga C. (2019). The neuroactive potential of the human gut microbiota in quality of life and depression. Nat. Microbiol..

[11] Engen P.A., Dodiya H.B., Naqib A., Forsyth C.B., Green S.J., Voigt R.M., Kordower J.H., Mutlu E.A., Shannon K.M., Keshavarzian A. (2017). The Potential Role of Gut-Derived Inflammation in Multiple System Atrophy. J. Park. Dis..

[12] Schepici G., Silvestro S., Bramanti P., Mazzon E. (2019). The Gut Microbiota in Multiple Sclerosis: An Overview of Clinical Trials. Cell Transplant..

[13] Boertien J.M., Pereira P.A., Aho V.T., Scheperjans F. (2019). Increasing Comparability and Utility of Gut Microbiome Studies in Parkinson’s Disease: A Systematic Review. J. Park. Dis..

[14] Klingelhoefer L., Reichmann H. (2015). Pathogenesis of Parkinson disease—The gut–brain axis and environmental factors. Nat. Rev. Neurol..

[15] Borody T., Rosen D., Torres M., Campbell J., Nowak A. (2011). Myoclonus-dystonia Affected by GI Microbiota? 940. Off. J. Am. Coll. Gastroenterol./ACG.

[16] Ma L., Keng J., Cheng M., Pan H., Feng B., Hu Y., Feng T., Yang F. (2021). Gut Microbiome and Serum Metabolome Alterations Associated with Isolated Dystonia. Msphere.

[17] Foster J.A., McVey Neufeld K.-A. (2013). Gut–brain axis: How the microbiome influences anxiety and depression. Trends Neurosci..

[18] Bercik P., Collins S.M., Verdu E.F. (2012). Microbes and the gut-brain axis. Neurogastroenterol. Motil..

[19] Strandwitz P. (2018). Neurotransmitter modulation by the gut microbiota. Brain Res..

[20] O’Mahony S.M., Clarke G., Borre Y.E., Dinan T.G., Cryan J.F. (2015). Serotonin, tryptophan metabolism and the brain-gut-microbiome axis. Behav. Brain Res..

[21] Agus A., Planchais J., Sokol H. (2018). Gut Microbiota Regulation of Tryptophan Metabolism in Health and Disease. Cell Host Microbe.

[22] Timmers E.R., van Faassen M., Smit M., Kuiper A., Hof I.H., Kema I.P., Tijssen M.A., Niezen-Koning K.E., de Koning T.J. (2021). Dopaminergic and serotonergic alterations in plasma in three groups of dystonia patients. Park. Relat. Disord..

[23] Smit M., Kuiper A., Han V., Jiawan V., Douma G., van Harten B., Oen J., Pouwels M., Dieks H., Bartels A. (2016). Psychiatric co-morbidity is highly prevalent in idiopathic cervical dystonia and significantly influences health-related quality of life: Results of a controlled study. Park. Relat. Disord..

[24] Timmers E.R., Smit M., Kuiper A., Bartels A.L., van der Veen S., van der Stouwe A.M., Santens P., Bergmans B., Tijssen M.A. (2019). Myoclonus-dystonia: Distinctive motor and non-motor phenotype from other dystonia syndromes. Park. Relat. Disord..

[25] Timmers E., Kuiper A., Smit M., Bartels A., Kamphuis D., Wolf N., Poll-The B., Wassenberg T., Peeters E., de Koning T. (2017). Non-motor symptoms and quality of life in dopa-responsive dystonia patients. Park. Relat. Disord..

[26] Kanehisa M., Sato Y., Kawashima M., Furumichi M., Tanabe M. (2015). KEGG as a reference resource for gene and protein annotation. Nucleic Acids Res..

[27] Alpizar-Rodriguez D., Lesker T.R., Gronow A., Gilbert B., Raemy E., Lamacchia C., Gabay C., Finckh A., Strowig T. (2019). *Prevotella copri* in individuals at risk for rheumatoid arthritis. Ann. Rheum. Dis..

[28] Pedersen H.K., Gudmundsdottir V., Nielsen H.B., Hyotylainen T., Nielsen T., Jensen B.A.H., Forslund K., Hildebrand F., Prifti E., Falony G. (2016). Human gut microbes impact host serum metabolome and insulin sensitivity. Nature.

[29] Li J., Zhao F., Wang Y., Chen J., Tao J., Tian G., Wu S., Liu W., Cui Q., Geng B. (2017). Gut microbiota dysbiosis contributes to the development of hypertension. Microbiome.

[30] Zalar B., Haslberger A., Peterlin B. (2018). The Role of Microbiota in Depression—A Brief Review. Psychiatr. Danub..

[31] Cox L.M., Maghzi A.H., Liu S., Tankou S.K., Dhang F.H., Willocq V., Song A., Wasén C., Tauhid S., Chu R. (2021). Gut Microbiome in Progressive Multiple Sclerosis. Ann. Neurol..

[32] Mortaş H., Bilici S., Karakan T. (2020). The circadian disruption of night work alters gut microbiota consistent with elevated risk for future metabolic and gastrointestinal pathology. Chronobiol. Int..

[33] Kaakoush N.O. (2020). Sutterella Species, IgA-degrading Bacteria in Ulcerative Colitis. Trends Microbiol..

[34] Kaper J.B., Nataro J.P., Mobley H.L.T. (2004). Pathogenic Escherichia coli. Nat. Rev. Microbiol..

[35] Martin-Gallausiaux C., Marinelli L., Blottiere H.M., Larraufie P., Lapaque N. (2021). SCFA: Mechanisms and functional importance in the gut. Proc. Nutr. Soc..

[36] Louis P., Flint H.J. (2017). Formation of propionate and butyrate by the human colonic microbiota. Environ. Microbiol..

[37] Aho V.T.E., Houser M.C., Pereira P.A.B., Chang J., Rudi K., Paulin L., Hertzberg V., Auvinen P., Tansey M.G., Scheperjans F. (2021). Relationships of gut microbiota, short-chain fatty acids, inflammation, and the gut barrier in Parkinson’s disease. Mol. Neurodegener..

[38] Unger M.M., Spiegel J., Dillmann K.-U., Grundmann D., Philippeit H., Bürmann J., Faßbender K., Schwiertz A., Schäfer K.H. (2016). Short chain fatty acids and gut microbiota differ between patients with Parkinson’s disease and age-matched controls. Park. Relat. Disord..

[39] Taras D., Simmering R., Collins M.D., Lawson P.A., Blaut M. (2002). Reclassification of Eubacterium formicigenerans Holdeman and Moore 1974 as Dorea formicigenerans gen. nov., comb. nov., and description of *Dorea longicatena* sp. nov., isolated from human faeces. Int. J. Syst. Evol. Microbiol..

[40] Pidcock S.E., Skvortsov T., Santos F.G., Courtney S.J., Sui-Ting K., Creevey C.J., Huws S.A. (2021). Phylogenetic systematics of Butyrivibrio and Pseudobutyrivibrio genomes illustrate vast taxonomic diversity, open genomes and an abundance of carbohydrate-active enzyme family isoforms. Microb. Genom..

[41] Yano J.M., Yu K., Donaldson G.P., Shastri G.G., Ann P., Ma L., Nagler C.R., Ismagilov R.F., Mazmanian S.K., Hsiao E.Y. (2015). Indigenous Bacteria from the Gut Microbiota Regulate Host Serotonin Biosynthesis. Cell.

[42] Roager H.M., Licht T.R. (2018). Microbial tryptophan catabolites in health and disease. Nat. Commun..

[43] Jinnah H.A., Neychev V., Hess E.J. (2017). The Anatomical Basis for Dystonia: The Motor Network Model. Tremor Other Hyperkinetic Mov..

[44] Baj A., Moro E., Bistoletti M., Orlandi V., Crema F., Giaroni C. (2019). Glutamatergic Signaling along the Microbiota-Gut-Brain Axis. Int. J. Mol. Sci..

[45] Busner J., Targum S.D. (2007). The clinical global impressions scale: Applying a research tool in clinical practice. Psychiatry (Edgmont).

[46] Consky E.S., Lang A.E., Jankovic J., Hallet M. (1994). Clinical Assessments of Patients with Cervical Dystonia.

[47] Burke R.E., Fahn S., Marsden C.D., Bressman S.B., Moskowitz C., Friedman J. (1985). Validity and reliability of a rating scale for the primary torsion dystonias. Neurology.

[48] Frucht S., Leurgans S.E., Hallett M., Fahn S. (2002). The Unified Myoclonus Rating Scale. Adv. Neurol..

[49] Van Faassen M., Bouma G., De Hosson L.D., Peters M.A., Kats-Ugurlu G., de Vries E., Walenkamp A.M., Kema I.P. (2019). Quantitative Profiling of Platelet-Rich Plasma Indole Markers by Direct-Matrix Derivatization Combined with LC-MS/MS in Patients with Neuroendocrine Tumors. Clin. Chem..

[50] van Faassen M., Bischoff R., Eijkelenkamp K., de Jong W.H.A., van der Ley C.P., Kema I.P. (2020). In Matrix Derivatization Combined with LC-MS/MS Results in Ultrasensitive Quantification of Plasma Free Metanephrines and Catecholamines. Anal. Chem..

[51] Yu Z., Morrison M. (2004). Improved extraction of PCR-quality community DNA from digesta and fecal samples. Biotechniques.

[52] Swarte J.C., Douwes R.M., Hu S., Vila A.V., Eisenga M.F., van Londen M., Gomes-Neto A.W., Weersma R.K., Harmsen H.J., Bakker S.J. (2020). Characteristics and Dysbiosis of the Gut Microbiome in Renal Transplant Recipients. J. Clin. Med..

[53] Swarte J.C., Li Y., Hu S., Björk J.R., Gacesa R., Vila A.V., Douwes R.M., Collij V., Kurilshikov A., Post A. (2022). Gut microbiome dysbiosis is associated with increased mortality after solid organ transplantation. Sci. Transl. Med..

[54] Gacesa R., Kurilshikov A., Vila A.V., Sinha T., Klaassen M.A.Y., Bolte L.A., Andreu-Sánchez S., Chen L., Collij V., Hu S. (2022). Environmental factors shaping the gut microbiome in a Dutch population. Nature.

[55] Silverman J.D., Roche K., Holmes Z.C., David L.A., Mukherjee S. (2019). Bayesian Multinomial Logistic Normal Models through Marginally Latent Matrix-T Processes. arXiv e-Prints.

