# Supplementary material for: Gut Microbiome Composition in Dystonia Patients
Source: Int J Mol Sci. 2023 Jan 25;24(3):2383. doi: 10.3390/ijms24032383 (PMC9916458; doi:10.3390/ijms24032383)
Supplement: Supplementary file 1 [file ijms-24-02383-s001.zip › ijms-2141766-supplementary.pdf]

## Supplementary material

**Supplementary Table S1.** Characteristics of used questionnaires

| | | |
|---|---|---|
| **Adults** | | |
| *Psychiatric co-morbidity* | Mini International Neuropsychiatric Interview—PLUS (MINI—PLUS) | Presence of a lifetime psychiatric disorder according to the DSM-IV |
| *Severity of depression* | Beck Depression Inventory (BDI) | Score reflecting depressive symptoms:<br>0 – 9: minimal<br>10–18: mild<br>19–29: moderate<br>30–63: severe |
| *Severity of anxiety* | Beck Anxiety Inventory (BAI) | Score reflecting anxiety:<br>0–9: normal or no anxiety, 10–18: mild to moderate,<br>19–29: moderate to severe 30–63: severe anxiety |
| *Severity of obsessive compulsive disorder (OCD)* | Yale–Brown obsessive compulsive scale (Y–BOCS) | Score reflecting OCD:<br>0 – 7: no OCD<br>8 – 15: mild OCD<br>15 – 25: moderate OCD<br>26 – 40: severe OCD |
| *Quality of sleep* | Pittsburgh Sleep Quality Index (PSQI) | Score range 0 – 21<br>Clinical relevant score ≥ 5 |
| *Fatigue* | Fatigue Severity Scale (FSS) | Score range 9 – 63<br>Clinical relevant score ≥ 36 |
| *Excessive daytime sleepiness* | Epworth Sleepiness Scale (ESS) | Score range 0 – 24<br>Clinical relevant score ≥ 8 |
| **Children** | | |
| *Psychiatric co-morbidity* | Mini International Neuropsychiatric Interview—KID (MINI—KID) | Presence of a lifetime psychiatric disorder according to the DSM-IV |
| *Severity of depression* | Children's Depression Inventory (CDI) | Score reflecting depression:<br>0 – 12: mild<br>12 – 16: moderate<br>> 16 severe |
| *Severity of anxiety* | Screen for Child Anxiety-Related Emotional Disorder (SCARED) | Score range 0 – 138<br>Clinical relevant score<br>7-12 years old >147<br>13-19 years old >131 |
| *Severity of obsessive-compulsive disorder (OCD)* | Child Yale–Brown obsessive compulsive scale (CY–BOCS) | Score reflecting OCD:<br>0 – 7: no OCD<br>8 – 15: mild OCD<br>15 – 25: moderate OCD<br>26 – 40: severe OCD |
| **Adults and children** | | |
| *Severity of depression* | z-score of BDI and CDI | Based on mean scores from healthy controls |
| *Severity of anxiety* | z-score of BAI and SCARED | Based on mean scores from healthy controls |

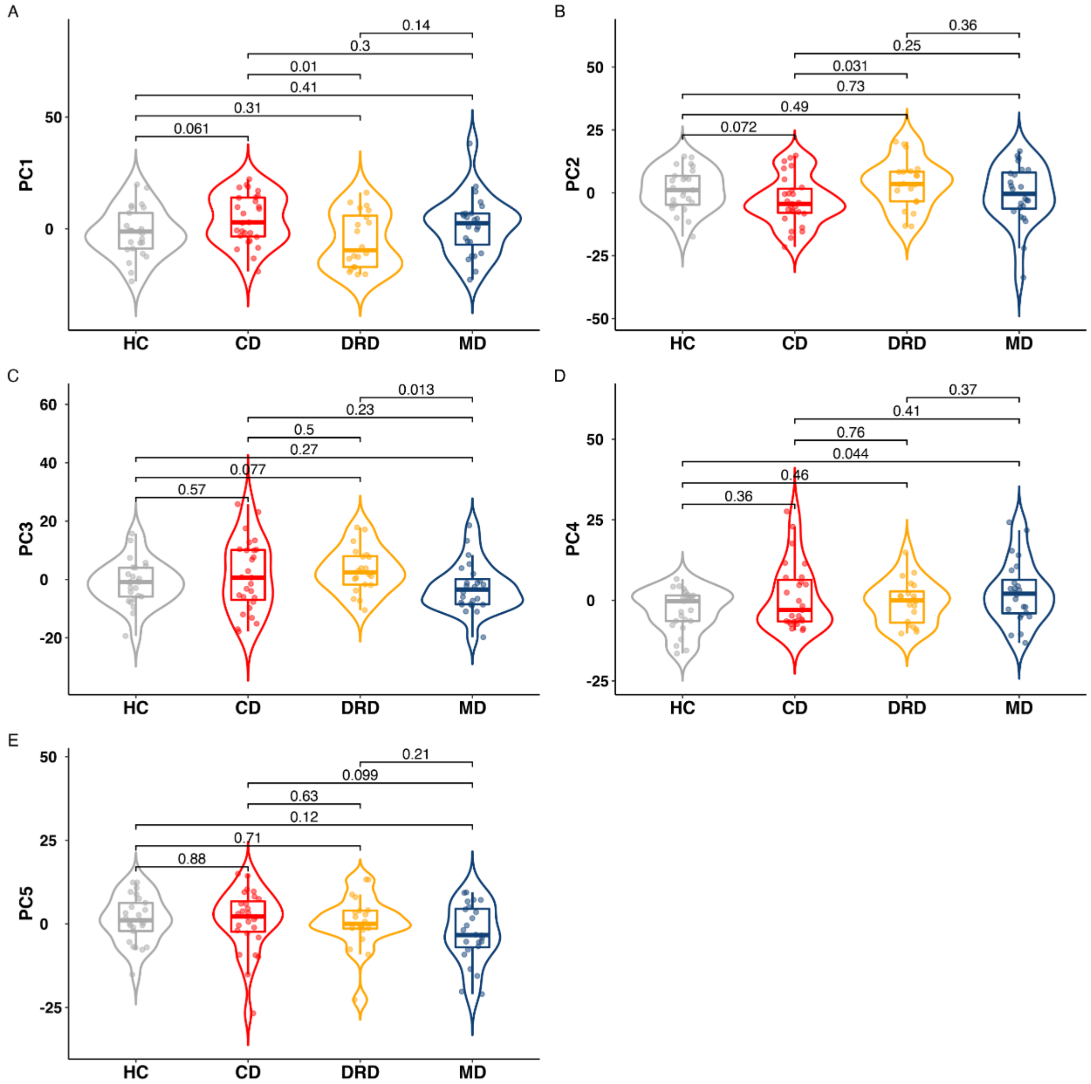


**Supplementary Figure S1**. Violin plots of the different principle components (PC) per group based on the Aitchison distance. p-values are calculated using paired Wilcoxon test. HC: healthy controls; CD: cervical dystonia; DRD: dopa-responsive dystonia; MD: myoclonus dystonia.

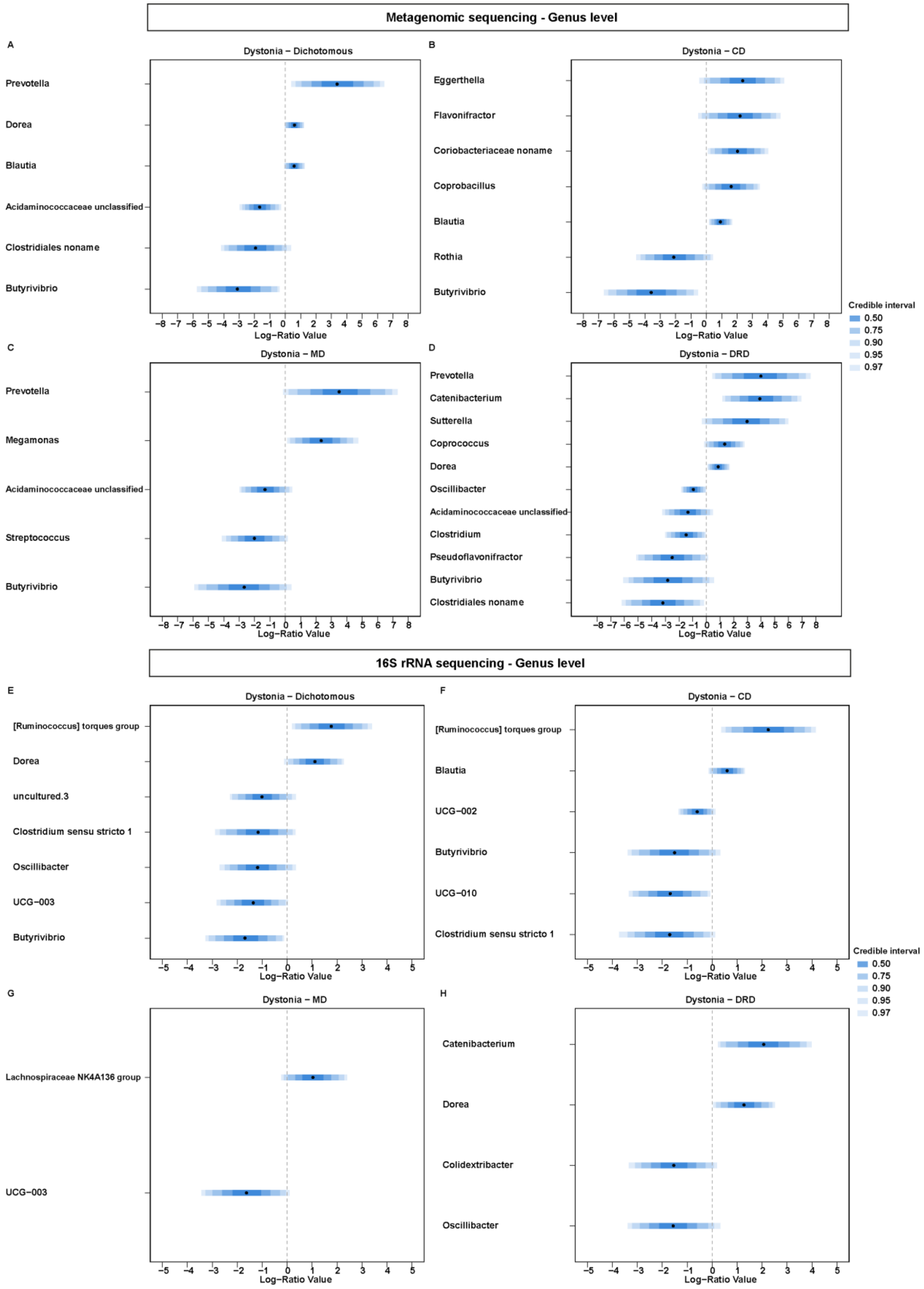


**Supplementary Figure S2.** Results of a pibble model (a Multinomial Logistic-Normal Linear Regression model) showing differences in abundance of the microbiota on a species level based on results of the metagenomic sequencing. A cut-off of 90% confidence interval was used. Plots

show the comparison between healthy controls and the whole dystonia group, cervical dystonia (CD), dopa-responsive dystonia (DRD) and myoclonus dystonia (MD) groups as depicted.

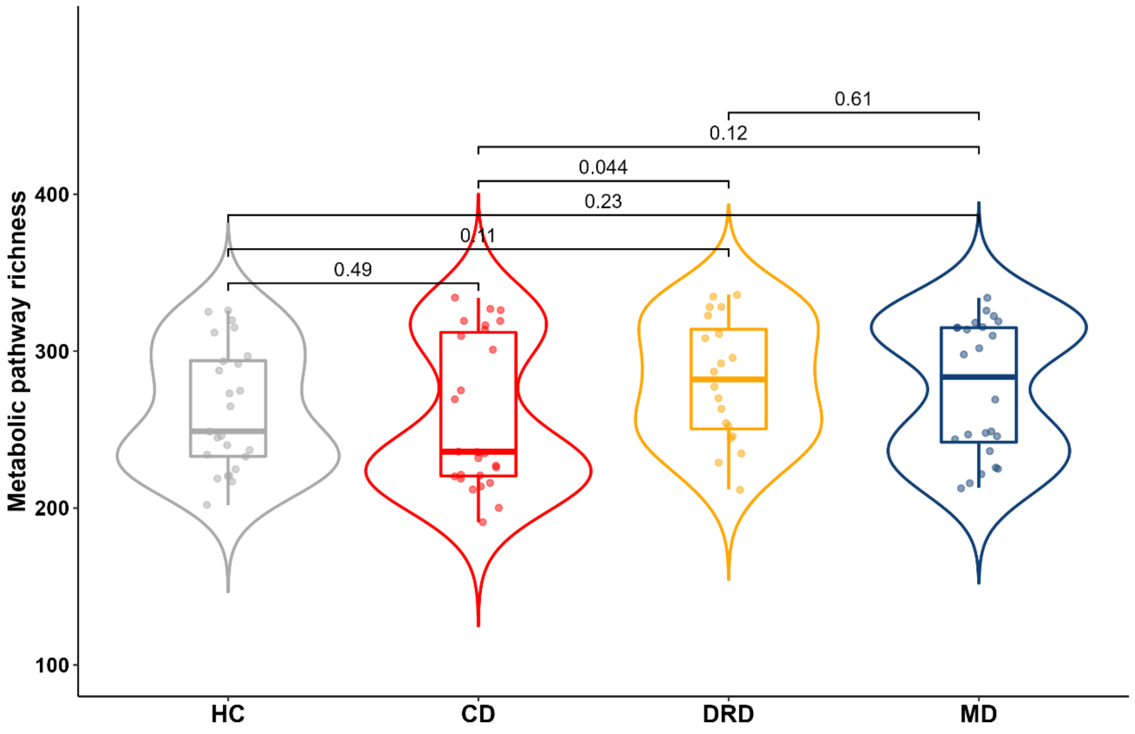


**Supplementary Figure S3.** Violin plot of the Shannon diversity index used to determine differences in metabolic pathway richness between the four groups. HC: healthy controls; CD: cervical dystonia; DRD: dopa-responsive dystonia; MD: myoclonus dystonia.

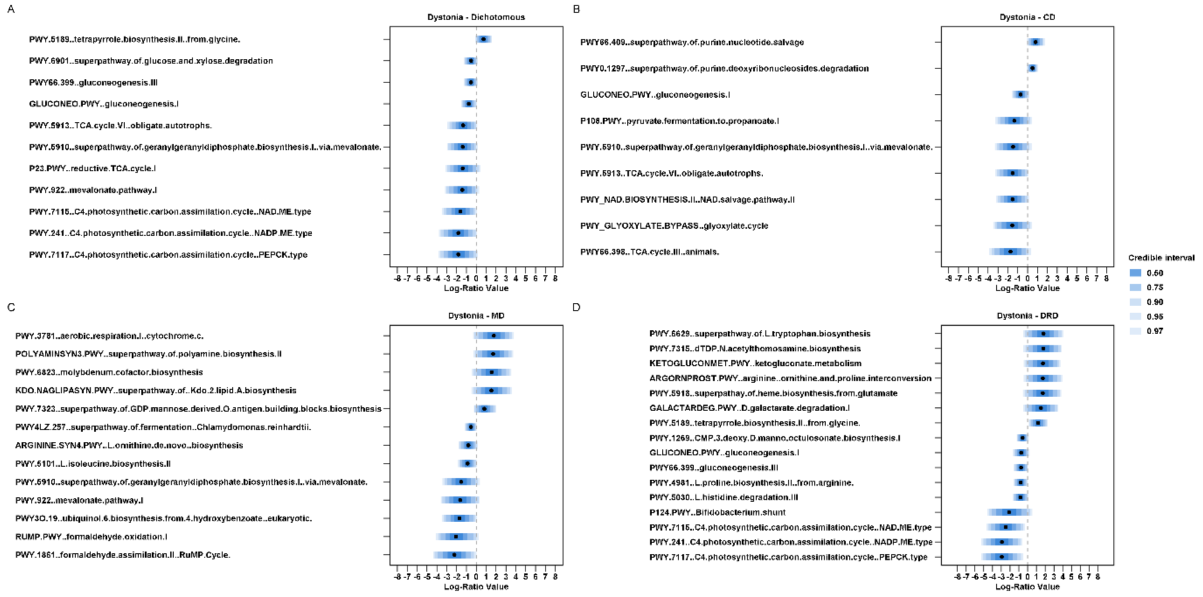


**Supplementary Figure S4.** Results of a pibble model (a Multinomial Logistic-Normal Linear Regression model) showing differences in abundance of metabolic pathways between dystonia patients and healthy controls based on results of the metagenomic sequencing. A cut-off of 90%

confidence interval was used. Plots show the comparison between healthy controls and the whole dystonia group, cervical dystonia (CD), dopa-responsive dystonia (DRD) and myoclonus dystonia (MD) groups as depicted.

## Supplementary results

To test whether the results on the species level metagenomics could be recapitulated with our 16S rRNA sequencing data, we performed the same analysis but on the genus level. When the whole dystonia group was compared to the healthy controls, a higher abundance of *Dorea* and a lower abundance of *Butyrivibrio* was found in both sequencing methods. In CD patients compared to healthy controls, a higher abundance of the *Blautia* genus and a lower abundance of *Butyrivibrio* were found. The *Catenibacterium* and *Dorea* genera were more abundant in the DRD group compared to the healthy controls (Figure 2C and Supplementary figure 2).
